# Modified Nanoclays/Straw Fillers as Functional Additives of Natural Rubber Biocomposites

**DOI:** 10.3390/polym13050799

**Published:** 2021-03-05

**Authors:** Justyna Miedzianowska, Marcin Masłowski, Przemysław Rybiński, Krzysztof Strzelec

**Affiliations:** 1 Institute of Polymer & Dye Technology, Lodz University of Technology, Stefanowskiego 12/16, 90-924 Lodz, Poland; marcin.maslowski@p.lodz.pl (M.M.); krzysztof.strzelec@p.lodz.pl (K.S.); 2 Institute of Chemistry, Jan Kochanowski University, Żeromskiego 5, 25-369 Kielce, Poland; przemyslaw.rybinski@ujk.edu.pl

**Keywords:** nanoclays, straw, natural rubber, modification, biocomposite

## Abstract

Increasingly, raw materials of natural origin are used as fillers in polymer composites. Such biocomposites have satisfactory properties. To ensure above-average functional properties, modifications of biofillers with other materials are also used. The presented research work aimed to produce and characterize elastomeric materials with a straw-based filler and four different types of montmorillonite. The main research goal was to obtain improved functional parameters of vulcanizates based on natural rubber. A series of composites filled with straw and certain types of modified and unmodified nano-clays in various ratios and amounts were prepared. Then, they were subjected to a series of tests to assess the impact of the hybrids used on the final product. It has been shown that the addition of optimal amounts of biofillers can, inter alia, increase the tensile strength of the composite, improve damping properties, extend the burning time of the material and affect the course of vulcanization or cross-linking density.

## 1. Introduction

Polymer composites currently play an essential role in the global production of industrial products. Extensive research conducted for decades proves that the addition of natural fillers to polymers allows both to improve the strength parameters of these materials, but also positively affects their acoustic and barrier properties, flammability, and aesthetics [1,2,3,4]. Technical progress has made it possible to successfully process such materials on an industrial scale, while reducing the consumption of fossil fuels on our planet. The ecological approach to the processing and production of polymer biocomposites directly contributes to a significant reduction in carbon dioxide emissions. By producing and using bio-renewable materials, the imbalance in the demand and supply of products made from non-renewable raw materials is prevented, waste management is balanced, and recycling problems are reduced [5,6].

Polymer composites with plant-derived fillers seem to be of particular interest, as they in many cases replace the previously used inorganic fillers [7]. The authors have been successfully developing the subject of elastomeric biocomposites containing cereal straw in their structure for several years, demonstrating its positive impact on the functional properties of the designed materials [8,9,10,11]. The straw that is post-harvest waste has a structure similar to that of wood, which has a decisive impact on obtaining materials with above-average mechanical properties. The use of straw as a filler in the production of composites is also advantageous due to the low cost of the final product compared to filling with other fibers of this type, such as jute. Each year, farms produce millions of tons of straw [12]. A huge percentage of this raw material is treated as waste [13]. Only a small part is used as litter or food for animals [14] or as an additive to fertilizers [15] or biofuels [16,17]. Nevertheless, it should be emphasized that despite the many positive aspects of using cereal straw as an additive to polymer composites, its operation can still be improved and intensified, for example, by selected modifications. One of them is modification with nanoadditives, carried out by physical grinding of ingredients and creating hybrid fillers. This technique has great potential in creating specific active functional compounds in the technology of composite materials.

The authors, in their works, focus primarily on the use of a specific, selected group of polymeric materials, which are elastomers. From the point of view of the technology of composites reinforced with natural fibers, these materials are underestimated and not fully recognized scientifically, unlike thermoplastics and resins [18,19]. Elastomers, taking into account the technological division and rheological properties, are one of the basic groups of polymers that show a high degree of elastic deformation with low stress. The main representative of this group is natural rubber—a polymer of plant origin [20]. It is obtained from caoutchouc plants, mainly *Hevea brasiliensis*, a Brazilian rubber tree [21]. The use of natural rubber in the technology of polymeric materials has been known for centuries, mainly due to its very good strength properties.

Due to the need to meet the constantly growing industrial, technological, scientific, and consumer requirements of polymer composites, it is necessary to use additional, innovative methods to improve their properties [6]. One of them is the modification of the fillers with the use of other, multifunctional materials, for example, aluminosilicates. Montmorillonite is a volcanic mineral [22,23]. It has a lamellar structure consisting of three layers that are interconnected. The outer layers are composed of tetrahedral silicon dioxide crystals, and the inner layer is the octahedral magnesium oxide or alumina crystals. Metal cations such as sodium, calcium or potassium are present between the layers. Montmorillonites are characterized by a wide range of applications such as: Pharmaceutical excipient in drug delivery systems [24], agents improving the rheological properties of drilling fluid [25], paints, cosmetics and lubricants [26], heavy oil and bitumen samples modifiers [27], organic nano-adsorbents in wastewater treatment [28], functional nanomaterials for clay/polymer nanocomposites [29,30,31]. The hydrophilicity of montmorillonite limits its affinity to most known polymers. The solution to this problem is its hydrophobization [32]. Currently, there are many methods known to modify the aluminosilicates. The most popular of them is the method that involves the introduction of various organic cations derived from aliphatic amino acids and alkyl ammonium salts with the longest aliphatic chain in place of sodium cations [33]. Due to amino acids, a proton is transferred from the -COOH group of an amino acid to its -NH_2_ group. This results in a cationic exchange between the (-NH^3+^) amino acid group and the Na^+^ and K^+^ cations of montmorillonite, as is the case with ammonium cations [34]. As a result of the above process, the surface properties of MMTs change from hydrophilic to hydrophobic. The penetration of organic cations between the individual layers of montmorillonite results in increasing the interlayer distances, which makes it possible for other organic compounds, e.g., monomers or polymers, to penetrate there as well [35].

The aim of the study was to create biocomposites with various fillers, including straw and modified or unmodified montmorillonites. Three different modified montmorillonites were selected as components of the produced hybrid fillers. Then, the impact of these hybrids on the final properties of the rubber product, such as mechanical properties under static and dynamic conditions, tear strength, hardness, damping properties, and flammability, was assessed. The article is a continuation of research on hybrid straw systems with the addition of nanofillers [36,37,38]. This research attempts to provide information on the effects of both physical and chemical modification of fillers on the elastomer matrix. The obtained results are promising and, most importantly, the proposed solutions have not been the subject of scientific considerations in the elastomer technology so far.

## 2. Materials and Methods

### 2.1. Materials

#### 2.1.1. Elastomer 

Natural Rubber (NR), ribbed smoked sheets—RSS 1 (Torimex-Chemicals Ltd. Sp. z o.o, Lodz, Poland).

#### 2.1.2. Biofillers

Cereal straw mechanically modified by:

- Pure Montmorillonite (MMT0).
- Montmorillonite modified 35–45 wt. % dimethyldialkyl (C14-C18) amine (MMT1).
- Montmorillonite modified 15–35 wt. % octadecylamine, 0.5–5 wt. % Aminopropyltriethoxysilane (MMT2).
- Montmorillonite modified with 25–30 wt. % methyl dihydroxyethyl hydrogenated ammonium tallow (MMT3).

#### 2.1.3. Sulfur Cross-Linking System

- Sulfur—S, (Siarkopol, Tarnobrzeg, Poland).
- Zinc oxide—ZnO, (Huta Bedzin, Poland).
- Mercatobenzothiazole—MBT, (Saint Louis, MO, USA).
- Stearic acid—SA, (Avantor Performance Materials, Gliwice, Poland).

Compositions of elastomer mixtures are presented in Table 1.

### 2.2. Methods

#### 2.2.1. Preparation of Biofillers 

**Figure 1:**
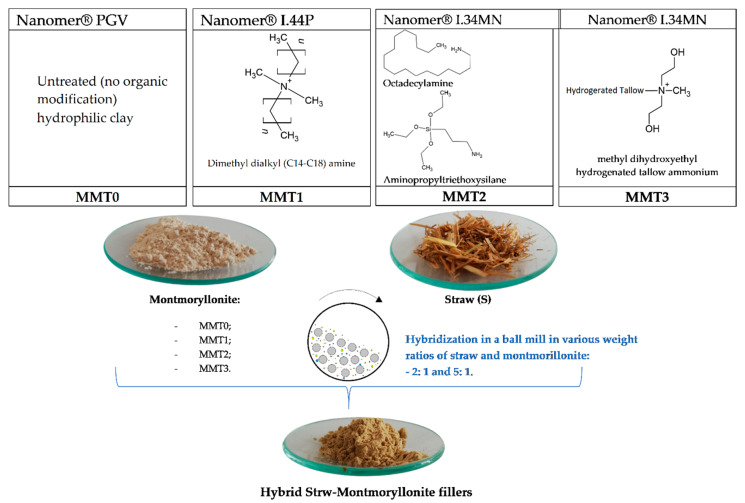
Biofillers modification scheme.

The cereal straw was milled with a Pulverisette 5 Classic Line planetary ball mill (Fritsch, Idar-Oberstein, Germany) (grinding time = 3 h, rotation speed = 300 rpm) to obtain a fine powder. The prepared cereal biofiller was dried at 70 °C for 48 h. Then the straw powder was modified by adding selected montmorillonites in two weight ratios 2:1 and 5:1 (straw:MMT). The modification was carried out with the use of a Pulverisette 5 planetary mill (Fritsch GmbH, Idar-Oberstein, Germany) for 15 min at 300 rpm. The filler preparation scheme is shown in Figure 1.

#### 2.2.2. Thermogravimetric Analysis

Thermal analysis of modified bioadditives was carried out using a TGA analyzer (Mettler Toledo, Greifensee, Switzerland). Temperature range 25–700 °C, with up to 600 °C measurements were made in argon atmosphere (55 mL/min), while from 600 °C to 700 °C in air (55 mL/min). The heating rate was 20 °C/min.

#### 2.2.3. Scanning Electron Microscopy Analysis 

The morphology of both fillers and composites was examined by scanning electron microscope (Hitachi, TM-1000, Tokyo Japan). Before SEM measurements, the samples were sputtered with carbon. The accelerating voltage was 25 kV.

#### 2.2.4. Contact Angle Measurements

Measurements of the water contact angle of the modified straw fillers were made on previously pressed discs. The tests were performed using an OCA 15EC Goniometer (DataPhysics Instruments GmbH, Filderstadt, Germany). About 10 µL (a drop) of distilled water was deposited on the smooth surface of the tablets and a photo was taken. On the basis of the drop shape analysis, the value of the occlusion angle was measured

#### 2.2.5. Flammability of Biofillers

The biofillers were examined by means of the PCFC (pyrolysis combustion flow calorimeter) from Fire Testing Technology Limited (East Grinstead, Great Britain). The temperature of the pyrolyzer was 750 °C, while that of the combustor 900 °C. During measurement the following parameters were recorded: Maximum heat emission rate (HRR_max_), total heat emitted (HR), and heat capacity. The sample was heated using a linear temperature program, and the volatile thermal degradation products were swept from the pyrolysis chamber by inert gas and combined with excess oxygen in a tubular furnace at the temperature of 900 °C to force complete combustion (oxidation) of the fuel. Combustion products CO_2_, H_2_O, and acid gases were scrubbed from the gas stream and the transient heat release rate was calculated from the measured flow rate and oxygen concentration after correcting for flow dispersion. The maximum (peak) value of the PCFC heat release rate normalized for the initial sample mass and heating rate was a material flammability parameter with units of heat release capacity (J/gK) which depends only on the chemical composition of the sample and is proportional to the burning rate of the material in a fire.

#### 2.2.6. Preparation of Biocomposites

In the first stage, a laboratory micromixer (Brabender, Duisburg, Germany) was used, and then a BRIDGE laboratory two-roll mill. The mixing of the ingredients in the micromixer was carried out at the speed of 40 rpm at the temperature of 50 °C and lasted for a total of 8 min. The first 4 min were devoted to the plasticization of natural rubber, and for the next 4 min, the actual mixing of the rubber with the biofiller took place. In the next stage, the prepared premixes were combined with a sulfur cross-linking system using a laboratory two-roll mill operating at room temperature. After mixing the ingredients, the produced rubber mixtures were flattened (sheets about 0.5 mm thick were obtained).

#### 2.2.7. Determination of Rheometric Properties of Rubber Mixtures

The rheometric properties of the prepared mixtures were tested with the MDR rheometer (Alpha Technologies, Hudson, OH, USA) at 160 °C. The values of the minimum (M_min_) and maximum (M_max_) torque were determined, as well as the optimal vulcanization time (*t*_90_). The torque increase ΔM for composites was calculated using the equation 1 [39]:

(1) $$\Delta M=M_{\max}\;-{\;M}_{\min}.\;$$

#### 2.2.8. Vulcanization of Rubber Mixtures

Steel vulcanization molds placed between heated shelves of a hydraulic press were used to vulcanize the rubber mixtures. Vulcanization took place at a temperature of 160 °C under a pressure of 15 MPa for 10 min.

#### 2.2.9. Determination of the Cross-Linking Deity

The study was carried out by determining the weight gain of the tested vulcanizate samples caused by the interaction of the solvent–toluene. The cross-linking density of composites was calculated using the Flory-Rehner Equation (2):

(2) $$\gamma_{e}=\frac{\ln\left(1\;-{\;V}_{r}\right)\;+{\;V}_{r}\;+\;\mu V_{r}^{2}}{V_{0}\left(V_{r}^{\frac{1}{3}}\;-\;\frac{V_{r}}{2}\right)}.\;$$

where:

$\gamma_{e}$—the cross-linking density (mol/cm^3^), V_0_—the molar volume of solvent (toluene: 106.7 cm^3^/mol). µ—for the interaction of natural rubber—toluene at 25 °C, the Huggins parameter is equal to [40]:

(3) $$\mu =0.478+0.228\cdot V_{r}$$

#### 2.2.10. Examination of the Mechanical Properties of Vulcanizates under Dynamic Conditions

The Ares G2 rheometer (TA Instruments, New Castle, DE, USA) was used to perform the dynamic-mechanical analysis (DMA). Discs with a diameter of approx. 25 mm and a thickness of approx. 2 mm were used for the tests. The samples were placed successively in the measuring gap and then subjected to the action of shear deformation increasing with time at a constant temperature and a constant strain frequency. As a result of the measurements, the curves of changes in the loss modulus (G′′) and the conservative modulus (G′) were observed as a function of the amplitude of the shear deformation of the tested materials. The Payne effect value (ΔG′) was calculated from the following Equation (5):

(4) $$\Delta \;G^{\prime }={G^{\prime }}_{0}\;-\;{G^{\prime }}_{\infty }.\;$$

where:

G′_0_—the value of the loss modulus obtained for the smallest applied amplitude of shear strain [MPa].

G′_∞_—the value of the loss modulus obtained for the highest applied shear strain amplitude [MPa].

#### 2.2.11. Examination of the Mechanical Properties of Vulcanizates under Static Conditions

Tensile strength and elongation at break tests were carried out using a universal testing machine (ZwickRoell, Ulm, Germany), following the PN-ISO 37 standard. Samples in the shape of type 3 paddles, about 1 mm thick and 4 mm wide, were used. The exact thickness of the individual samples was determined with an accuracy of 0.01 mm using a thickness gauge. From each type of biocomposite, 5 samples were tested and the results were averaged. Measurements were carried out at a tensile speed of 500 mm/min at room temperature. The research allowed the determination of the following parameters:

- Tensile strength (T_S_) [MPa];
- Relative elongation at break (E_B_) [%].

#### 2.2.12. Hardness Determination

The hardness test was carried out in accordance with the PN-ISO 868 standard using the Shore A hardness tester (ZwickRoell, Ulm, Germany), with a digital measuring system. Disc-shaped vulcanizates with a thickness of about 17.8 mm and a diameter of 35 mm were used. Each sample was tested at six different measuring points and the obtained results were averaged.

#### 2.2.13. Damping Properties of Vulcanizates

The study of the damping properties of vulcanizates was carried out with the use of a universal testing machine (ZwickRoell, Ulm, Germany). Cylindrical samples with a diameter of 35 mm and a thickness of about 17.8 mm were used. The samples were successively subjected to compressive stresses ranging from 0 to 0.7 MPa, after which the stress was reduced to zero. Three measurement cycles were performed for each sample. The result of the measurement was the strain-stress relationship in the form of a hysteresis loop. On their basis, the values of the difference between the compression work and the work during deformation reduction (∆W_i_) and the compression work (W_ibel_) were calculated. The values of the relative attenuation coefficient were calculated based on the following Equation (4) [41]:

(5) $$T\tau w=\;\frac{\Delta W_{i}}{W_{\mathrm{ibel}}}\;\cdot 100\;\left[\%\right]$$

#### 2.2.14. Flammability of Biocomposites

The flammability of biocomposites was determined by a cone calorimeter (Fire Testing Technology Ltd., East Grinstead, UK). Composite samples with dimensions of (100 × 100 ± 1) mm and thickness of (2 ± 0.5) mm were tested in a horizontal position with a heat radiant flux density of 35 kWm^−2^. During the tests, the following parameters were recorded: Initial sample weight; time to ignition (TTI); sample weight during testing; total heat released (THR); effective combustion heat (EHC); average weight loss rate (MLR); heat release rate (HRR); and final sample weight.

## 3. Results and Discussion

### 3.1. Thermogravimetric Analysis of Fillers

**Figure 2:**
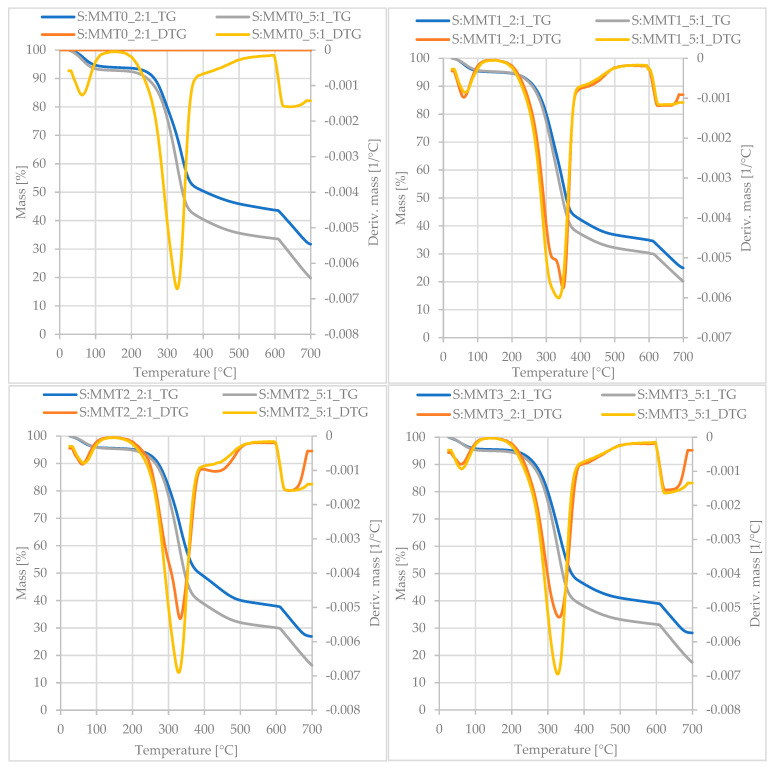
The thermogravimetric (TG) curves and the derivative of the TG versus time (DTG) curve of straw-montmorillonite fillers.

Thermal properties of straw-montmorillonite fillers were determined by the method of thermogravimetric analysis (TGA). Figure 2 shows the thermogravimetric (TG) curves and the derivative of the TG curve as a function of time (DTG). Besides, Table 2 summarizes the parameters read from the thermal curves, such as 5% (T_5_) and 50% (T_50_) weight loss temperature, sample weight loss at 100 °C (L_100_) and the residue at 700 °C (R_700_).

All straw-montmorillonite fillers were characterized by a four-stage DTG curve. First, free water evaporated from the tested samples, which resulted in the appearance of a peak in the temperature range of 50–100 °C. The S:MMT0 filler samples were characterized by the highest water content, by the determined weight loss at 100 °C (L_100_). This is due to the greater hydrophilicity of the material and the ability to absorb moisture from the environment. The next stage was characterized by the greatest loss of mass and intensity, and it took place in the temperature range of 190–400 °C. Such a large loss of mass recorded on the TG curves was related to the overlapping of the distribution of several filler components. At these temperatures, the lignocellulosic material decomposed, thus the material of straw plant tissues, which is the main component of the filler. Hence, the weight loss and the intensity of the decomposition process in this range were greater for fillers with a higher proportion of straw, where its ratio to montmorillonites was 5:1. Considering the lignocellulosic material, hemicellulose is the easiest to undergo thermal degradation, its pyrolysis takes place at a temperature of 220–300 °C. Lignin is the least thermally stable and its intensive decomposition takes place in a wide temperature range (170–600 °C). In turn, the pyrolysis of cellulose takes place mainly at the temperature of 315–400 °C [42,43]. Also, there was a loss of interlayer water at this stage. Pugazhenthi et al. Claim that in the range 230–440 °C thermal decomposition of the organic nanoclay modifier took place. At this stage, there was also a 50% weight loss of the sample. The samples containing straw and unmodified montmorillonite were characterized by the highest T_50_ temperature (Table 2). This is because the applied modifiers were decomposed, thus reducing the thermal stability of the straw-modified montmorillonite systems. The third stage that can be distinguished on the DTG curve occurred in the temperature range of 400–500 °C (Figure 2). In this case, the distribution was more intensive for fillers with a higher proportion of montmorillonite (2:1). This is a consequence of the dehydroxylation of aluminum silicate [44]. Above 600 °C, the gas was changed and measurement was performed in an air atmosphere. Access to oxygen caused the appearance of the fourth peak on the DTG curve related to the combustion of the organic part formed after the pyrolysis process. Fillers containing more montmorillonite were characterized by a greater residue after thermal analysis (R_700_), which is related to a higher proportion of inorganic compounds in hybrid fillers (Table 2). Moreover, a greater weight loss was demonstrated by filler systems with modified nanoclay, as the organic modifiers included in the sample were degraded.

### 3.2. Measurement of the Contact Angle

Measuring the wettability of powders is a difficult task due to the liquid absorption during the experiment. Various methods of determining the contact angle of poured powders have been described in the literature, including measurements of wettability using the Washburn-like equation method [45,46]. Using a more common technique (e.g., static contact angle, Wilhelmy balance) on compacted powder samples [47]. The above-mentioned method of static measurement of the water contact angle of the surface of compressed straw and montmorillonite-based fillers were used in the study. Despite some limitations of this method, it makes it possible to determine and compare the hydrophilicity/hydrophobicity of the filler. It is an extremely important parameter that plays an important role in obtaining the appropriate adhesion between the filler and the rubber. Table 3 presents the obtained images of water drops on the surface of bioadditives tablets.

The filler consisting of cereal straw and unmodified montmorillonite (MMT0) showed the smallest contact angle (CA). This is a consequence of the hydrophilic nature of both components of the filler. Both straws as a lignocellulosic material and nanoclay are characterized by high hydrophilicity. Since nanoclays are natural cation exchangers, they can replace inorganic cations with organic ions. By introducing modifiers between the layers and into the air of montmorillonite, the hydrophobicity of the straw-modified montmorillonite systems was reduced. Increasing the hydrophobicity of the filler makes them compatible with polymer matrices [48]. The highest contact angle was obtained for the straw-montmorillonite system modified with dimethyldialkyl (C14-C18) amine (S: MMT1). The reduction of the hydrophilicity of the bioadditive is due to the introduction of long carbon chains with hydrophobic properties into montmorillonite. The other straw fillers containing modified montmorillonites were also characterized by increased hydrophobic properties. The ratio of ingredients hybridized with each other turned out to be equally essential. The increased proportion of montmorillonite had a positive effect on increasing the value of the contact angle, making the filler more compatible with the polymer matrix. This is most likely because straw, as a natural lignocellulosic material, is more hydrophilic than montmorillonite. Hence, a higher nanoadditive content in the filler resulted in an increased contact angle. This effect was particularly noticeable when the modified nanoclays were applied. Due to the non-polar character of natural rubber, fillers with greater hydrophobicity interact more effectively with macromolecules of polymer chains.

### 3.3. Morphology of Hybrid Fillers

**Figure 3:**
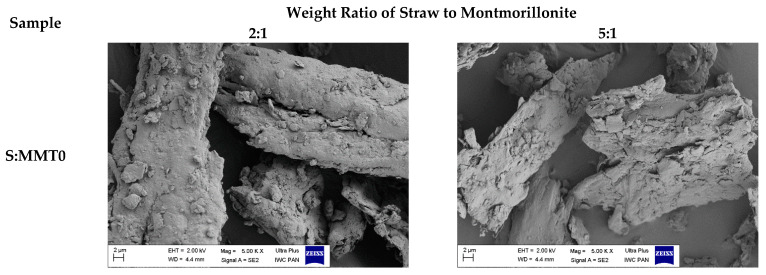

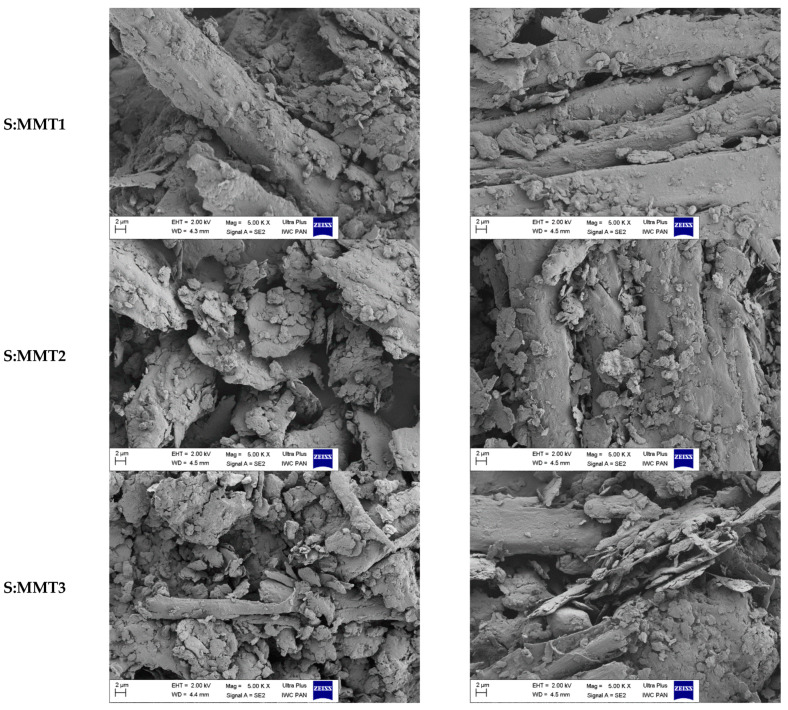
SEM image of straw-montmorillonite.

Figure 3 Illustrates SEM images of fillers formed as a result of the hybridization of cereal straw with nanoclays. Their analysis shows that the straw has been crushed into irregular particles, often elongated like fibers. Montmorillonite particles were characterized by a flat shape. The dimensions of such montmorillonite plates were about a few micrometers in diameter and a few nanometers in thickness. In the case of fillers which included unmodified montmorillonite (MMT0), straw and nanoclay created a more homogeneous structure. On the other hand, fillers containing modified nanoadditives produced looser systems. Separated particles of montmorillonites and straw were visible in the filler. Flat-shaped and elongated filler particles can play an important role in reducing the flammability of composites. Particularly important is the layered structure of montmorillonites, which reduces the possibility of air diffusion into the sample during its combustion. At the same time, it reduces the release of volatile products of destruction. In turn, taking into account the reinforcement of the material, longitudinal natural fibers may have a positive effect of improving mechanical properties. Additionally, the applied nanoparticles, also modified, can intensify the reinforcement of the rubber material.

### 3.4. Flammability of Biofillers

The test results obtained by the PCFC microcalorimetry method (pyrolysis combustion flow calorimeter) indicated the low flammability of the prepared biofiller. The lowest value of the HRR parameter (heat release rate), as well as the THR (total heat release) and HRC (heat release capacities) parameters, were found in the mixture of cereal straw with unmodified montmorillonite. Chemical modification of aluminosilicate, regardless of the type of modifier used, aimed at increasing intercalation, or possibly exfoliation of montmorillonite in the elastomer matrix, did not significantly reduce the flammability of the biofiller obtained for the tests. Taking into account the HRR, THR and HRC parameters, all tested bio-filler samples, i.e., S:MMT0, S:MMT1, S:MMT2, and S:MMT3, were characterized by a comparable level of flammability. For example, the value of the HRR parameter for the sample S:MMT0 (2:1) was 98.64 W/g, for S:MMT1 105.5 W/g, for S:MMT2 97.1 W/g, and for S:MMT3 111.8 W/g (Table 4).

The increase in cereal straw content in the prepared filler from 2 to 5 parts by weight, regardless of the montmorillonite modification method, increased its flammability. The higher values of the HRR, THR, and HRC parameters of the bio-filler 5:1 to 2:1 resulted directly from the increase (% by mass) of the carbon component content in the mass of the filler. However, it should be emphasized that along with the increase in the amount of cereal straw in the mass of the bio-filler, regardless of the method of montmorillonite modification, the value of the THRR parameter increased. In the case of the S:MMT1 filler, the difference between the 2:1 and 5:1 weight ratio was 24 °C, in the case of the S:MMT2 filler, the difference was 8 °C, and for the S:MMT3 filler 18 °C. The increase in the THRR parameter value for the S:MMT 5:1 bio-filler clearly indicated an increase in the thermal stability of the sample during its thermal decomposition. This was undoubtedly due to the insulating nature of the carbonate filler formed during the decomposition of the sample.

### 3.5. Rheometric Properties and Vulcanization Time

**Figure 4:**
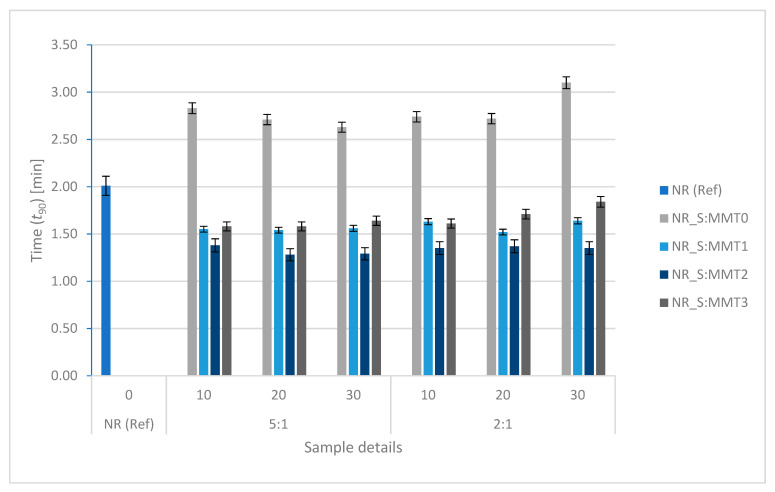
The optimum curing time (*t*_90_) for natural rubber composites.

The minimum M_min_ and the maximum M_max_ values of the torques were determined from the rheometric curves of the rubber mixtures. Moreover, the increase in torque during the cross-linking process (ΔM) was calculated. The values of these parameters are summarized in Table 5. Moreover, the optimal cross-linking time (*t*_90_) was determined as 90% of the time in which the maximum torque is obtained (Figure 4). As a result, it was possible to obtain preliminary information on the influence of the biofillers used on the properties of the mixtures and the characteristics of the cross-linking process.

The obtained values of the maximum torque (M_max_) allow stating that the increase of this parameter with the reference mixture was influenced by the addition of the tested biofillers, regardless of their type. The M_max_ value also increased with the increase of the filling degree of the mixtures, which was related to the increase in their stiffness. Much higher values of M_max_ were obtained for rubber mixtures containing straw filler hybridized with modified montmorillonites.

The values of the minimum torque M_min_ for mixtures containing unmodified montmorillonite MMT0 were similar or even lower than for the reference sample. Analyzing the results obtained for the mixtures with other montmorillonites, it could be noticed that the M_min_ values were higher compared to the reference sample and in most cases they maintained an increasing tendency along with the increasing filling degree of the mixtures, which in turn was related to their increasing viscosity. Moreover, mixtures containing straw:montmorillonite filler in a weight ratio of 2:1 were characterized by a higher M_min_ value. The high content of non-filler in the composite increased in viscosity. The highest M_min_ values were obtained for the mixtures containing the filler based on montmorillonite MMT3, which means that they were characterized by the highest viscosity. A consequence of the higher viscosity of the blend can be a high degree of dispersion of the filler in the polymer matrix.

As can be seen from the data presented in the table, in the case of mixtures with the addition of each of the fillers used, the value of the torque increase was higher than for the reference sample, which was unfilled natural rubber. It can also be seen that along with the increasing content of biofiller, an increase in the torque gain (ΔM) of the mixtures was observed. The type of montmorillonite included in the filler had a decisive influence on the obtained ΔM values. The application of additives containing modified nanoclays increased the values of the torque increments during crosslinking. Among these composites, the highest values of this parameter were recorded for mixtures filled with MM1 and MM2 hybridized straw. As the increase in torque during vulcanization is an indirect measure of cross-linking density, it can be assumed that composites will be characterized by the most developed spatial structure. In these cases, in addition to the formation of covalent cross-links resulting from the sulfur vulcanization reaction, the filler–polymer interactions may play an important role. Ammonium and silane functionalized montmorillonites (in the case of MMT2) may show increased adhesion to the elastomer matrix and contribute to the creation of additional physical network nodes. Moreover, in the case of modified montmorillonites, due to the increase in the distance between the layers caused by the penetration of organic cations containing long alkyl chains, the amount of bonded rubber influencing the interaction of the matrix with the filler increases.

The application of straw-montmorillonite additives caused significant changes in the cross-linking kinetics of natural rubber composites. The results of the optimal cross-linking time obtained for the tested materials are presented in Figure 4.

The shortening of the optimal vulcanization time of composites containing straw filler with MMT1 and MMT2 could result from the fact that the amine groups present on the surface of modified montmorillonites could improve the efficiency of the cross-linking reaction. It is known that the basic environment of this type of reaction accelerates the curing processes and influences the efficiency of cross-linking. In the case of a composite containing a filler, which includes montmorillonite modified with methyl dihydroxyethyl hydrogenated tallow ammonium, the effect of reducing the *t*_90_ value could be the result of the quaternary ammonium compound acting as a surfactant [49], increasing the filler dispersion degree [50].

### 3.6. Vulcanizates Cross-Linking Density

**Figure 5:**
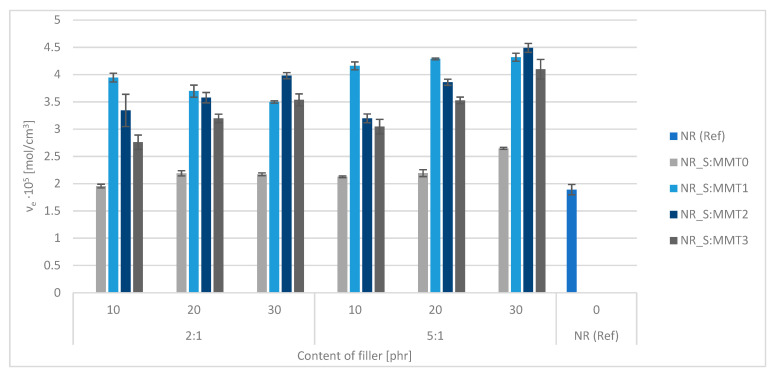
Cross-linking densities (ν_e_) of the tested vulcanizates containing straw fillers with the addition of montmorillonites.

Measurement of the equilibrium swelling of composites in toluene was carried out to check the influence of individual biofillers and their content on the lattice density value in the tested materials. The results of the experiment are depicted in Figure 5.

In composites with the participation of the filler, the value of the network density of vulcanizates increased with the increase of their filling level. The ν_e_ parameters obtained for the filler based on unmodified montmorillonite (MMT0) were the lowest and differed from the results obtained for other filled composites. Instead, they were the closest to the reference system.

Taking into account the ratio of straw to montmorillonite in the bioadditive, it was observed that the higher proportion of straw in the fillers (straw:montmorillonite ratio of 5:1) increased the value of ν_e_. It can be assumed that with the straw to nanoclay ratio of 2:1 the concentration of MMT nanoparticles was too high and the phenomenon of agglomeration took place. Agglomerates could reduce the number of physical network nodes due to their reduced activity.

The data contained in Figure 5 proved that in the case of all filled composites, regardless of the type and amount of biofiller used, the concentration of effective ν_e_ chains was higher than that obtained for the unfilled sample. The applied modifiers MMT1, MMT2 and MMT3 proved to be substances increasing the efficiency of cross-linking, which was manifested by an increase in cross-linking density. Moreover, all of the montmorillonite modifiers used in the structure had long alkyl chains which could penetrate between the layers present in the aluminosilicates and spread them apart during processing [51]. Consequently, the resulting voids facilitate the occlusion of the rubber. A layer of immobilized (so-called occluded or otherwise bound) rubber was formed around the filler, which had a significant impact on the structure of the spatial network of composites and the subsequent mechanical properties of the composites.

The mixtures with MMT1-based filler were characterized by the highest cross-linking density, which corresponded to the highest values of the increase in torque recorded during rheometric measurements.

### 3.7. Morphology of Biocompisites

**Figure 6:**
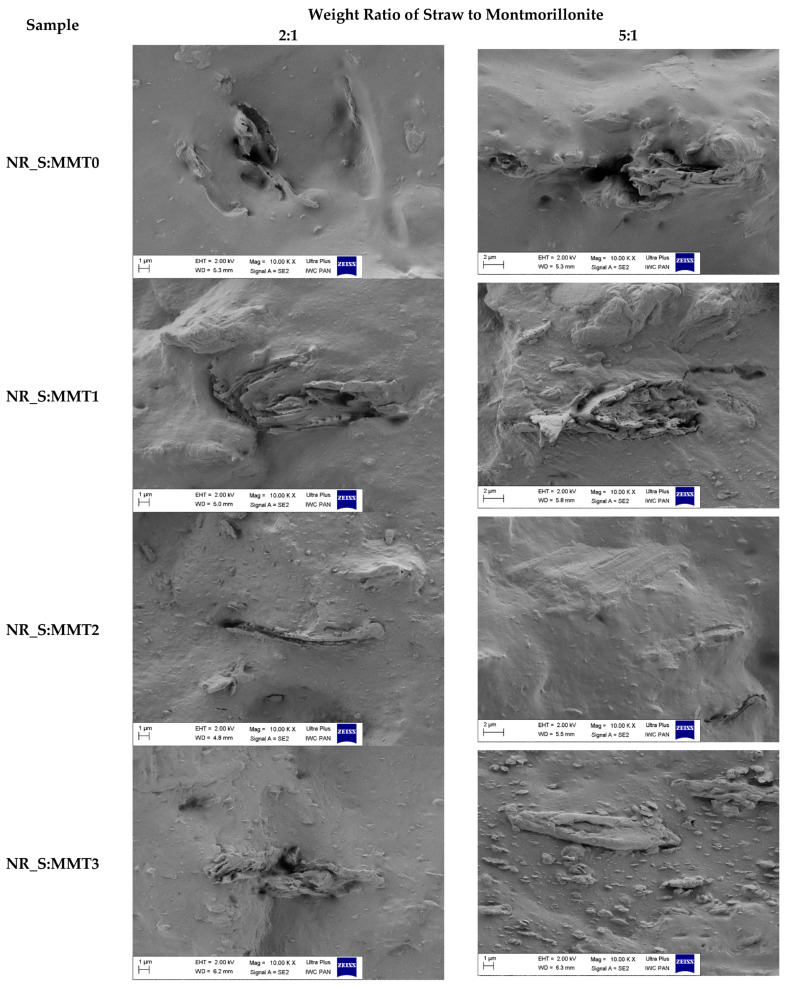
SEM images of biocomposites filled with 30 phr of straw-montmorillonite additives.

The morphology of the natural rubber composites was presented based on SEM images of vulcanizate breakthroughs containing 30 phr fillers (Figure 6). The biggest difference in the profiles of these materials can be noticed when comparing the structure of composites containing unmodified montmorillonite with other vulcanizates. In the NR_S: MMT0 composites, it was observed that the filler was locally clustered into larger aggregates and agglomerates. On the SEM images of these vulcanizates, straw particles were visible, while the montmorillonite particles most likely clumped around the straw fibers or created separated filler clusters. On the other hand, composites containing straw and modified montmorillonites in their structure, apart from straw fibers, in the matrix produced a well-dispersed network of nanoclay. The SEM images of the fractures of these vulcanizates contained particles of planar montmorillonites layered throughout the composite. This effect was especially visible for the composite designated NR_S:MMT3. The filler could be observed in the entire mass of elastomer, not only in larger clusters. Such composite morphology may have a particularly positive effect on the improvement of mechanical properties and reduction of flammability of materials.

### 3.8. Mechanical Properties of Biocomposites under Dynamic Conditions

**Figure 7:**
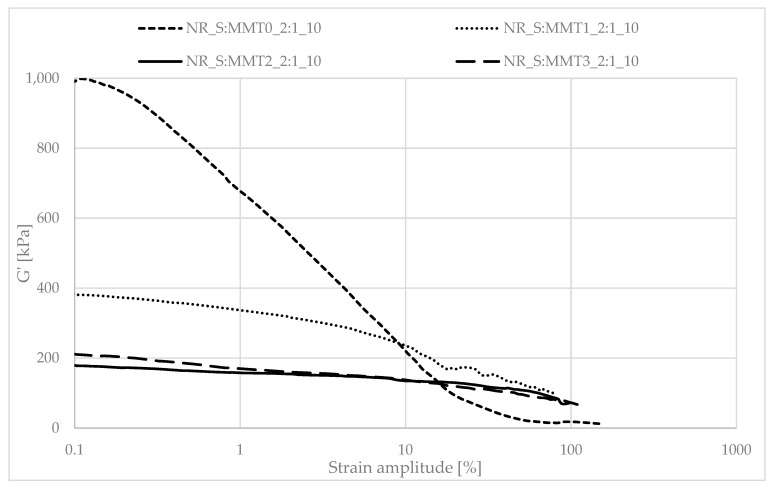
Dependence of the storage module as a function of the strain amplitude for natural rubber composites filled with straw:montmorillonite filler (10 phr), 2:1 ratio.

**Figure 8:**
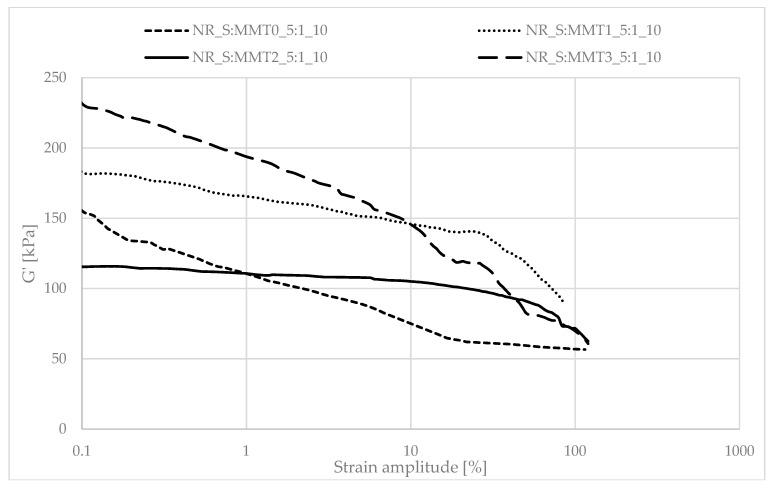
Dependence of the storage module as a function of the strain amplitude for natural rubber composites filled with straw:montmorillonite filler (10 phr), 5:1 ratio.

**Figure 9:**
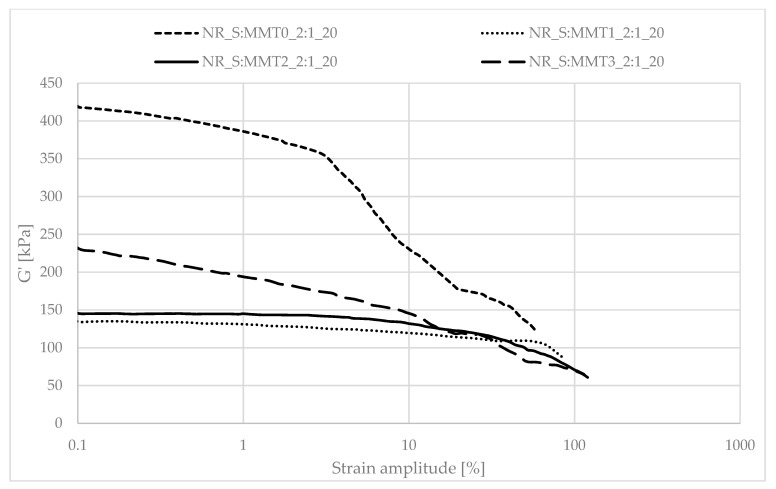
Dependence of the storage module as a function of the strain amplitude for natural rubber composites filled with straw:montmorillonite filler (20 phr), 2:1 ratio.

**Figure 10:**
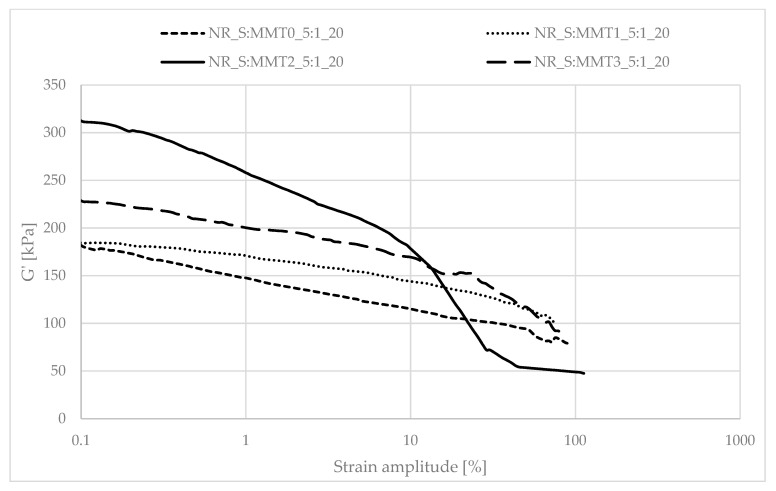
Dependence of the storage module as a function of the strain amplitude for natural rubber composites filled with straw:montmorillonite filler (20 phr), 5:1 ratio.

**Figure 11:**
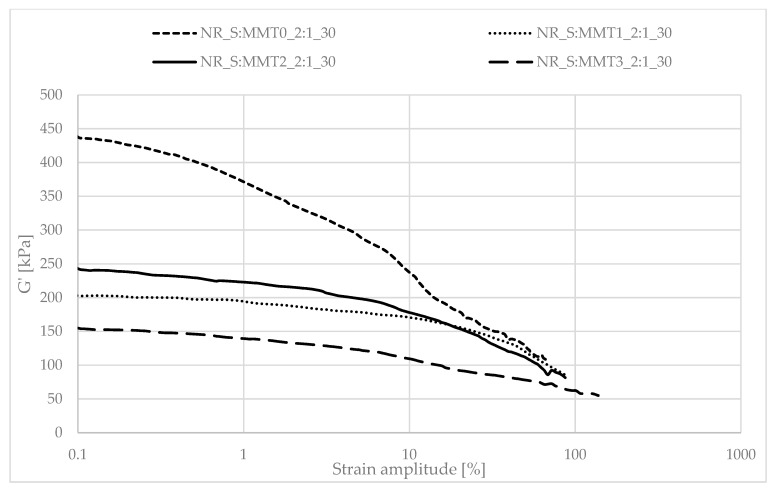
Dependence of the storage module as a function of the strain amplitude for natural rubber composites filled with straw:montmorillonite filler (30 phr), 2:1 ratio.

**Figure 12:**
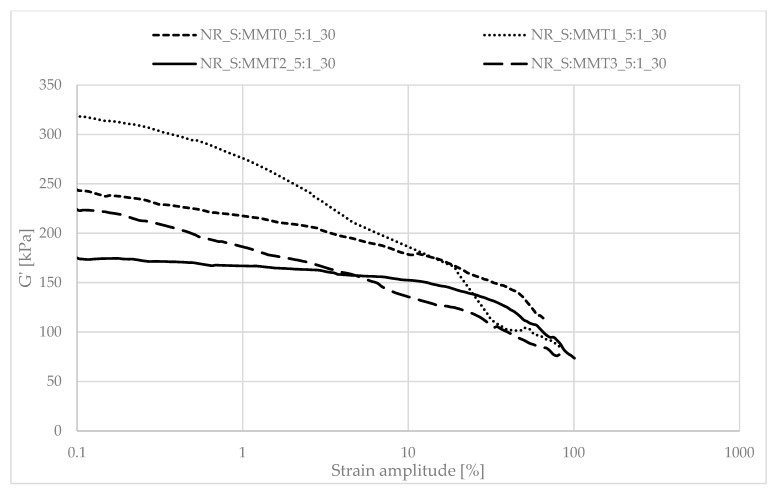
Dependence of the storage module as a function of the strain amplitude for natural rubber composites filled with straw:montmorillonite filler (30 phr), 5:1 ratio.

Determining the mechanical properties of composites under dynamic conditions is associated with the registration of the decrease in the storage module (G′) during dynamic deformation, related to the existence of the Payne effect. It depends, inter alia, on the existence and degree of violation of the secondary structure, formed with the participation of the filler particles, during the deformation of the tested samples. The Payne effect is typically attributed to deformational fracture and the re-creation of weak physical bonds that bind filler agglomerates together. It is therefore regarded as a measure of the filler microdispersion. A characteristic feature of the described effect is the fact that it occurs only in the case of vulcanizates, which include active fillers, hence its occurrence is related to filler-filler or filler–polymer interactions. A large decrease in the loss modulus during the dynamic-mechanical analysis of elastomer composites proves the expanded structure of the filler in the polymer matrix, which is the result of the activity of the additive. However, it should be mentioned that too high a drop in G′ as a function of deformation is the result of agglomeration and aggregation of filler particles in the rubber manifested by excessive filler-filler interactions. Figure 7, Figure 8, Figure 9, Figure 10, Figure 11 and Figure 12 show the effect of the type of filler used on the course of the G′ curves as a function of the strain amplitude.

The above charts show a decrease in the value of the conservative modulus G′ of the tested materials with an increase in the strain amplitude, regardless of the type of biofiller used. This dependence confirmed the existence of the Payne effect, which is related to the destruction of the filler microstructure in the elastomeric medium during dynamic measurements.

The addition of fillers influenced the decrease of the loss modulus in a variety of ways during dynamic mechanical analysis. The greatest decrease in G′ was recorded for the filler samples containing straw and unmodified montmorillonite in a weight ratio of 2:1 (NR_S:MMT0_2:1). The high content of nanofillers in addition could cause an increased tendency to agglomeration and aggregation affecting the filler-filler interactions, consequently leading to the achievement of high G′_max_ and Payne Effect. However, such a phenomenon is disadvantageous from the point of view of the subsequent performance of the material. In the case of the remaining straw-nonoclay systems in a weight ratio of 2:1, nanoadditive modifiers could contribute to the improvement of the dispersion of the entire filler. In particular, the quaternary ammonium compounds used as MMT2 and MMT3 organomodifiers can act as surfactants and improve the dispersion of fillers in the biocomposite (Effects of a quaternary ammonium salt on the properties of carbon-black-filled natural rubbercompounds). Consequently, it could have contributed to obtaining smaller decreases in the storage modulus during the study.

The composites containing the straw:montmorillonite 5:1 fillers had lower Payne Effects than the fillers (Figure 8, Figure 10 and Figure 12), where the ratio of these two components was 2:1 (Figure 7, Figure 9 and Figure 11). Nevertheless, these values were still high, which could indicate a good dispersion of the filler in the elastomer matrix and the active action of the bio-additives used.

### 3.9. Mechanical Properties of Composites under Static Conditions

The interactions between the filler and the polymer matrix have a major influence on the mechanical properties of rubber materials. An equally important phenomenon determining the mechanical strength is the transfer of stresses in the filler-polymer system. Depending on the amount, type, and various parameters of the filler used, such as, particle size, surface energy or interactions in the filler-polymer systems, they may occur to a greater or lesser extent. These interactions act as network nodes and therefore are a complementary element of the vulcanizate spatial network. The second type of interaction, i.e., filler-filler, is also very important, through which their spatial network is formed. It depends on the distance between the filler particles and their interactions.

**Figure 13:**
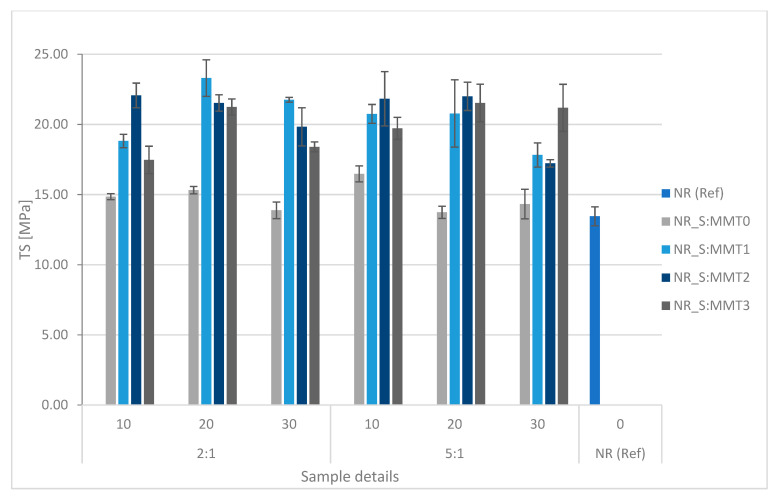
Influence of the type of biofiller and its content on the tensile strength values of vulcanizates.

The influence of the type and amount of used fillers on the mechanical properties of vulcanizates was investigated. Stress values at 100%, 200%, and 300% elongation (SE_100_ SE_200_ SE_300_) and elongation at break (E_b_) are presented in Table 6, and the obtained tensile strength (TS) values are graphically presented in Figure 13.

In the case of all tested filled biocomposites, an increase in tensile strength was noted in comparison with the reference system, which was the unfilled sample (Figure 13). Analyzing the results for the additives with MMT0-containing filler, i.e., unmodified montmorillonite, it can be noticed that the differences compared to the reference sample are insignificant. In this case, the addition of the filler did not have a large impact on the mechanical properties of the composite. Much more significant changes were observed after the application of straw bioadditives hybridized with modified montmorillonites. Each of these systems contributed to the strengthening of natural rubber, which increased in TS value. In particular, the enhancing effect can be attributed to fillers involving MMT1 and MMT2. The reinforcing effect of the fillers used was also confirmed by the increase in modules at low strains of 100%, 200%, and 300% compared to the reference system and composites marked with NR_S:MMT0. The use of organic modifiers based on ion exchange resulted not only in increased hydrophilicity, but also increased the interlayer spacing, which again allowed the polymeric chains to enter into the clay galleries. This phenomenon improved the compatibility of the filler with the matrix, and thus promoted better adhesion, resulting in an improvement in the mechanical strength of the composites. It is worth noting that the amount of straw in the filler did not significantly change the tensile strength of the vulcanizates. Biocomposites showed the highest tensile strength at the content of 20 phr of filler, higher content (30 phr) in almost every case caused a decrease in TS value. This was probably the effect of increased particle agglomeration leading to premature failure of the sample. This conclusion was confirmed by the values of the relative elongation at break. For each of the biocomposites with the highest filler content, a sudden decrease in the E_b_ value was observed (Table 6). 

The addition of fillers to the rubber had an ambiguous effect on the parameter of the elongation at break. All systems with MMT0-based filler increased E_b_ compared to the reference system. The E_b_ parameters obtained for the samples with the addition of straw fillers—modified montmorillonite were lower than the elongation at break noted for the samples containing S:MMT0. Low values of this parameter may have been due to the high cross-linking density. As a consequence, the flexibility of the samples was reduced. Besides, a part of the rubber that could penetrate between the interlayer spaces of the modified montmorillonites was immobilized, which resulted in a lower elongation at break such vulcanizates.

### 3.10. Hardness of Vulcanizates

**Figure 14:**
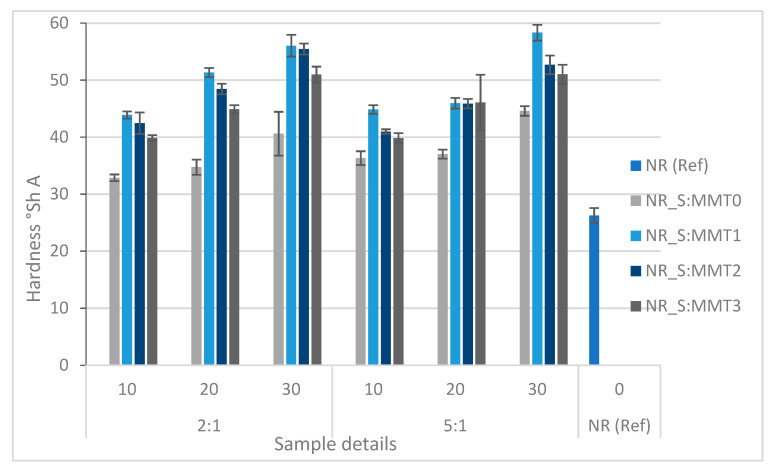
Influence of the type of biofiller and its content on the hardness values of composites at the straw:montmorillonite ratio 2:1.

An important parameter determining the hardness of the rubber vulcanizate is the degree of cross-linking of the sample and the amount of filler used. The obtained values of the hardness tested on the Shore A scale are presented in Figure 14.

As predicted, the hardness values of the tested biocomposites filled with hybrid fillers were higher than the reference sample. In the groups of individual fillers with a given ratio of straw to montmorillonite, the hardness value increased with the amount of filler used. Much higher hardness was recorded for composites containing straw-modified montmorillonite fillers. It was the effect of the increased density of these composites as a consequence of increased adhesion between the polymer and the filler. Moreover, the higher content of nanoclays in the filler (2:1) resulted in an increase in hardness compared to bio-additives where the straw to montmorillonite ratio was 5:1. The difference in hardness between these composites was less noticeable when the highest amount of filler was used—30 phr.

### 3.11. Vulcanizates Damping Properties

**Figure 15:**
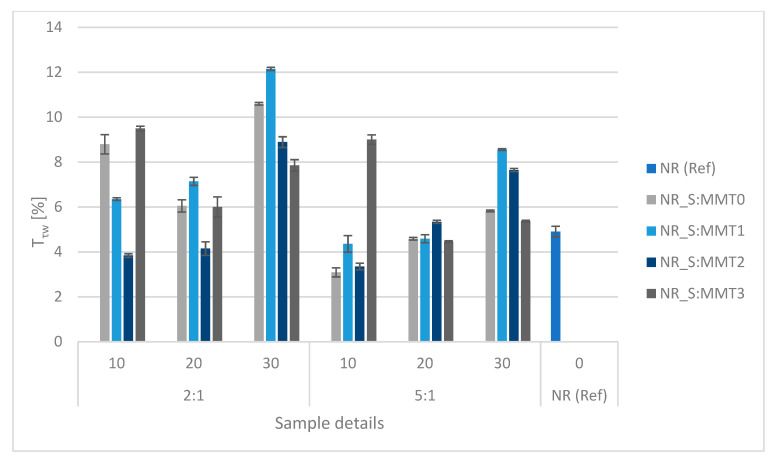
Influence of the type of biofiller and its amount on the damping properties of biocomposites.

Damping is another parameter that enables the assessment of the suitability of biocomposites in the plastics industry. Elastomeric materials can accumulate a large amount of energy used to deform them and to dissipate them partially in the form of heat. By comparing the value of dissipated energy and the value of energy used to deform the tested material, its ability to suppress deformation can be determined. Figure 15 shows the results of the study of the damping properties of natural rubber biocomposites. 

Based on the obtained relative damping values under compressive stresses (T_τw_), it can be concluded that the addition of MMT0 containing fillers in the straw:montmorillonite ratio of 2:1 made the damping properties much more intense than in the case of the reference system (NR (Ref)) and increased with the increase in the amount of filler used. However, at the ratio of 5:1, they were similar to the properties of the reference sample, and also showed an increasing tendency with the degree of filling. A similar situation occurred in the case of composites filled with straw and MMT1 montmorillonite. A different tendency was observed in the case of the filler containing montmorillonite MMT2. Relative damping values for both straw to montmorillonite ratios (2:1 and 5:1) increased with an increasing amount of filler used in biocomposites. However, for 10 and 20 phr both at 2:1 and 5:1 straw:montmorillonite ratios were lower than for the reference frame. Only at the amount of filler of 30 phr they reached the values of 8.89% and 7.65%, respectively. For fillers based on montmorillonite MMT3, regardless of the amount and ratio, T_τw_ values were higher than those corresponding to the reference sample. The highest relative damping values were obtained for samples containing 30 phr of the fillers S:MMT1_2:1 and S:MMT0 2:1 and they were respectively 12.15% and 10.60%.

### 3.12. Flammability of Biocomposites

**Figure 16:**
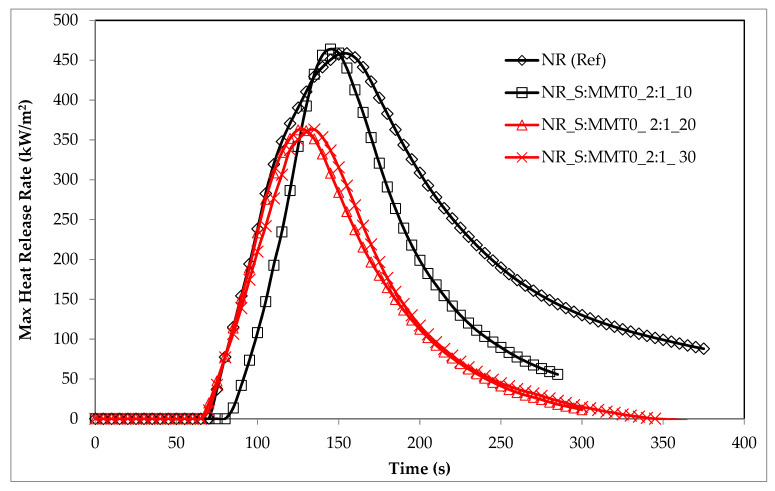
The heat release rate (HRR) max parameter of biocomposites filled with S:MMT0_2:1.

The flammability results of natural rubber biocomposites, obtained under real fire conditions (cone calorimeter), clearly indicated the anti-pyretic effect of the S: MMT bio-filler (Table 4). A significant reduction in flammability expressed by the parameters HRR, HRR_MAX_, THR, FIGRA, or MARHE was achieved with the filler content in the elastomer matrix of at least 20 parts by weight (Table 7 and Figure 16). For example, the filler S:MMT0 (2:1) introduced into the natural rubber matrix in the amount of 30 phr reduced its flammability expressed in the following parameters: HRR_MAX_, THR, FIGRA, or MARHE by: 20.8%, respectively; 44.2%; 9.15% and 32.15%. The bio-filler S:MMT0 introduced into the NR rubber matrix did not significantly affect the ignition time (*t*_i_), as well as the sample combustion time (*t*_f-o_), and the dynamics of its thermal decomposition and combustion (MLR, MLR_MAX_) (Table 7). The antipyretic effect of the S: MMT filler resulted, inter alia, from the tubular effect well described in the literature. The montmorillonite plates dispersed in the NR rubber matrix enable the diffusion of gaseous waste products into the flame only through strictly defined channels, at the same time constituting a barrier to oxygen diffusion inside the composite [52,53].

**Figure 17:**
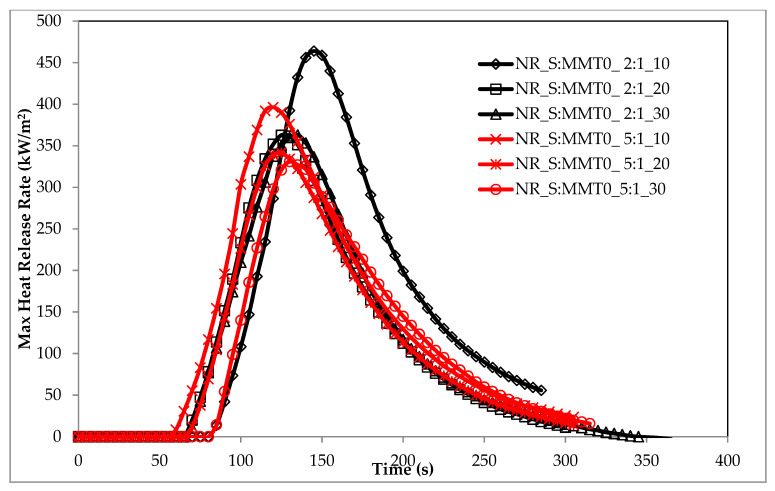
The HRRmax parameter of biocomposites filled with S:MMT0.

**Figure 18:**
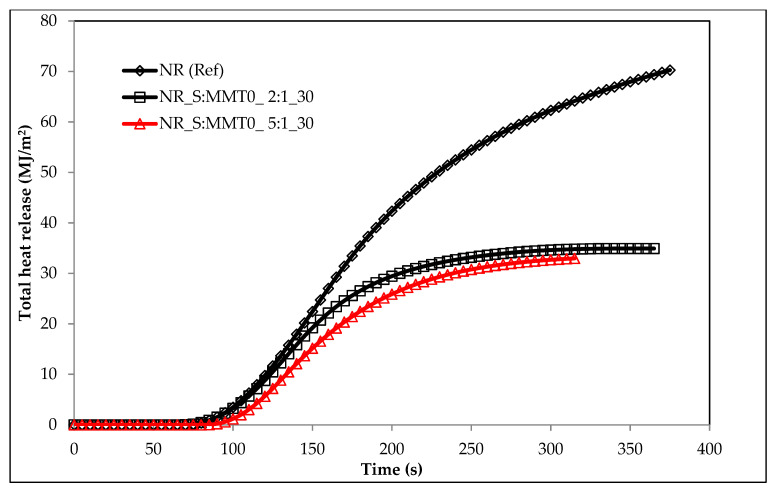
The total heat released (THR) parameter of biocomposites filled with 30 phr of S:MMT0.

The data in Table 7 also clearly showed that the composites containing the S:MMT 5:1 filler were characterized by lower flammability compared to the composites filled with S:MMT 2:1, which led to believe that the cellulose filler in the form of cereal straw had a decisive influence on the reduction of flammability of the tested composites (Figure 17 and Figure 18). During the decomposition of a composite containing S:MMT 5:1, carbonization reactions resulting in an insulating, uniform carbon layer occur more intensively than in the case of composites containing the S:MMT 2:1 filler. The review of the source literature clearly shows that during the decomposition of lignocellulose in the temperature range ΔT = 250–400 °C, as a result of dissociation of ether bonds and carbon-carbon, phenolic rings are formed, which are responsible for the increase in the amount of carbon residue with insulating properties [54].

Modification of MMT with the use of organic compounds influenced the antipyretic efficiency of the S:MMT filler. The best effect in the fire suppression process was recorded for the S:MMT3 5:1 filler. The NR rubber composite containing 30 parts by weight of the same filler was characterized by a 32% reduced value of the HRR_MAX_ parameter concerning the cross-linked NR rubber (Table 7) and a 51.3% reduction of the THR parameter. FIGRA and MARHE parameters were reduced by 8.81 and 35% compared to NR (Ref). The reduction of the fire hazard parameters of composites containing a bio-filler containing modified MMT directly resulted from its better distribution in the polymer matrix. The increase in the homogenization of the elastomer mixture, including the more effective intercalation of montmorillonite plates in the NR matrix, resulted not only in increasing the thermal stability (insulation) of the produced composites, but also in reducing the amount of liquid destructs generated during their decomposition, which was responsible for the transfer of fire, and thus for the intensification of processes burning.

**Table 7:** Flammability characteristics of biocomposites.

Sample Name	Filler Content [phr]	Weight Ratio of Straw to Montmorillonite	*t*_i_ (s)	*t*_f-o_ (s)	HRR (kW/m^2^)	HRR_max_ (kW/m^2^)	*t*HRR_max_ (s)	THR (MJ/m^2^)	EHC (MJ/kg)	EHC_max_ (MJ/kg)	MLR (g/s)	MLR_max_ (g/s)	AMLR (g/m^2^ × s)	FIGRA (kW/m^2^s)	MARHE (kW/m^2^)
**NR (Ref)**	0	-	60	287	264.3	458.5	155	60.2	33.4	56.9	0.07	0.265	18.21	2.95	218.3
**NR_S:MMT0**	10	2:1	75	214	256.1	463.9	145	35.2	18.7	74.8	0.12	0.287	21.97	3.19	167.4
	20		53	239	173.3	362.7	125	32.0	17.2	74.6	0.08	0.288	19.87	2.90	146.8
	30		61	261	168.7	363.1	135	33.6	18.3	78.6	0.08	0.280	19.92	2.68	148.1
**NR_S:MMT0**	10	5:1	49	233	196.1	396.3	120	36.1	18.7	70.6	0.09	0.308	19.3	3.3	166.4
	20		55	250	160.4	341.3	125	31.5	16.6	74.7	0.08	0.296	20.10	2.73	139.4
	30		74	246	161.3	330.9	130	30.5	16.8	78.8	0.09	0.337	21.00	2.53	129.6
**SNR_S:MMT1**	10	2:1	56	261	186.9	404.5	120	38.2	20.5	78.6	0.08	0.293	17.63	3.37	162.3
	20		48	209	206.8	380.4	115	32.7	18.7	70.1	0.09	0.321	20.30	3.30	163.3
	30		50	252	151.4	294.4	115	30.9	17.6	74.1	0.07	0.409	20.8	2.56	137.2
**NR_S:MMT1**	10	5:1	56	269	199.8	411.8	120	41.6	23.8	77.2	0.07	0.287	16.31	3.43	170.5
	20		50	194	210.6	394.9	115	30.1	17.3	76.9	0.10	0.305	20.00	3.43	159.8
	30		49	226	174.8	335.6	115	31.2	16.6	69.3	0.09	0.307	21.86	2.91	149.5
**NR_S:MMT2**	10	2:1	52	270	209.2	483.1	115	45.1	24.2	64.9	0.07	0.261	19.72	4.20	195.8
	20		53	212	213.2	408.8	120	33.7	19.1	75.3	0.09	0.289	20.55	4.02	163.7
	30		50	181	202.9	337.4	120	26.9	15.3	79.9	0.11	0.304	18.87	2.81	149.6
**NR_S:MMT2**	10	5:1	64	203	259.5	461.4	135	35.6	21.4	59.8	0.10	0.344	18.41	3.41	177.8
	20		43	206	183.5	339.5	115	30.0	18.1	59.4	0.08	0.279	17.92	2.95	152.0
	30		41	211	184.7	330.0	115	31.1	17.9	78.2	0.09	0.245	17.41	2.86	153.1
**NR_S:MMT3**	10	2:1	59	239	245.2	488.1	125	43.9	23.0	79.1	0.09	0.327	19.25	3.90	192.5
	20		52	213	226.7	432.4	115	35.9	21.3	77.2	0.09	0.292	21.09	3.76	176.9
	30		44	209	199.2	377.7	120	32.6	19.9	73.3	0.08	0.291	18.98	3.14	163.3
**NR_S:MMT3**	10	5:1	54	265	178.4	416.1	120	37.7	22.5	70.2	0.07	0.339	20.62	3.46	167.5
	20		52	255	184.2	387.6	125	37.0	19.8	77.1	0.08	0.315	21.36	3.10	163.3
	30		41	215	171.5	309.9	115	29.3	16.1	74.5	0.09	0.272	19.46	2.69	141.7

Where: *t*_i_—time to ignition, *t*_f-o_—time to flameout, HRR—heat release rate, HRR_max_—maximum heat release rate, *t*HRR_max_—time to maximum heat release rate, THR—total heat release, EHC—effective heat of combustion, EHC_max_—maximum effective heat of combustion, MLR—mass loss rate, MLR_max_—maximum mass loss rate, AMLR—average mass loss rate, FIGRA—HRR_max_/*t*HRR_max_, MARHE—maximum average heat release rate.

## 4. Conclusions

Hybrid fillers based on the straw with the addition of modified montmorillonites showed higher thermal stability in the lower temperature ranges of 100–300 °C. However, at higher temperatures (300–500 °C), they decomposed earlier. The contact angle studies confirmed the influence of modifiers present in nanoclays on the surface character of straw systems. Moreover, the changes caused by incorporation of the modified MMT into the straw were also visible from scanning electron microscopy images. The test results obtained by the microcalorimetry method confirmed the low flammability of the prepared biofiller.

The use of additives containing modified nanoclays increased the value of torque gain during cross-linking. The highest values of this parameter were recorded for mixtures filled with MMT1 and MMT2 hybridized straw. The applied straw/modified nanoclays systems turned out to be substances increasing the effectiveness of cross-linking, and these composites were characterized by the most developed spatial structure. Moreover, the use of straw–montmorillonite additives caused significant changes in the kinetics of cross-linking of natural rubber composites, significantly shortening this process. Dynamic mechanical analysis proved that in the produced biocomposites, regardless of the additive used, an extensive secondary structure of the filler was formed, which was confirmed by the existence of the so-called Payne effect. Furthermore, the enhancing effect of hybrid fillers has also been confirmed based on static mechanical tests. In particular, it was highlighted for vulcanizates filled with the straw:MMT1 and straw:MMT2 systems. All produced materials were characterized by high hardness. Taking into account the damping properties, it should be emphasized that the highest damping efficiency was achieved by vulcanizates with the highest content of biocomponents in the rubber matrix. The flammability results of natural rubber biocomposites, obtained under real fire conditions, clearly indicate the anti-pyretic effect of the S:MMT bio-filler.

## Figures and Tables

**Table 1:** Composition of elastomer mixtures.

Sample	Straw:MMT	Filler Content (phr)	NR (phr)	S (phr)	MBT (phr)	SA (phr)	ZnO (phr)
Unfilled NR	-	0	100	2	2	1	5
Straw:MMT0	2:1	10	100	2	2	1	5
		20					
		30					
	5:1	10	100	2	2	1	5
		20					
		30					
Straw:MMT1	2:1	10	100	2	2	1	5
		20					
		30					
	5:1	10	100	2	2	1	5
		20					
		30					
Straw:MMT2	2:1	10	100	2	2	1	5
		20					
		30					
	5:1	10	100	2	2	1	5
		20					
		30					
Straw:MMT3	2:1	10	100	2	2	1	5
		20					
		30					
	5:1	10	100	2	2	1	5
		20					
		30					

**Table 2:** Thermal parameters determined for biofillers: 5% (T_5_) i 50% (T_50_) weight loss temperature, weight loss of the sample at 100 °C (L_100_), residue at 700 °C (R_700_); standard deviations: T_5_ ± 1 °C, T_50_ ± 2 °C, L_100_ ± 2%, R_700_ ± 2%.

Sample of Filler	Weight Ratio of Straw to Montmorillonite	T_5_ [°C]	L_100_ [%]	T_50_ [°C]	R_700_ [%]
S:MMT0	2:1	92	5.4	407	32
	5:1	76	6.6	343	20
S:MMT1	2:1	162	4.6	359	25
	5:1	180	4.3	347	20
S:MMT2	2:1	207	4.1	386	27
	5:1	193	4.1	345	16
S:MMT3	2:1	203	4.2	366	28
	5:1	145	4.7	341	17

**Table 3:** Images of water drops on the surface of pressed fillers with determined contact angles (CA), standard deviations CA ± 5°.

Sample	Weight Ratio of Straw to Montmorillonite	
	2:1	5:1
**S:MMT0**	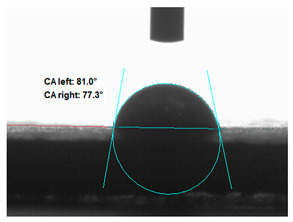	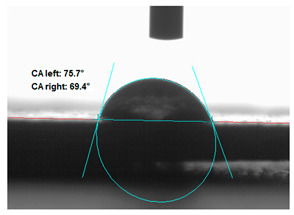
**S:MMT1**	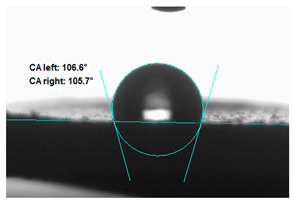	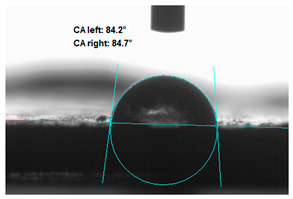
**S:MMT2**	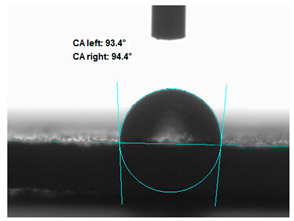	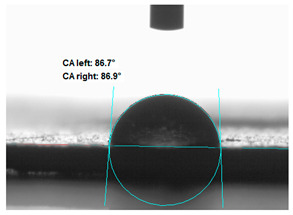
**S:MMT3**	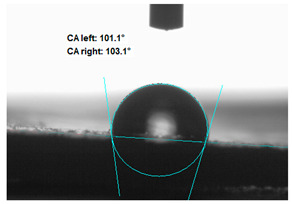	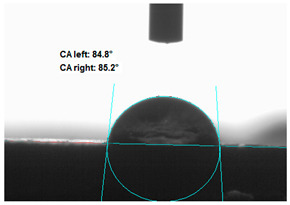

**Table 4:** Flammability results of fillers determined with a microcalorimeter; standard deviations HRRW/g, THRR ± 3 °C THR ± 0.5 kJ/g and HRC ± 3 J/gK.

Sample of Filler	Weight Ratio of Straw to Montmorillonite	HRR [W/g]	THRR [°C]	THR [kJ/g]	HRC [J/gK]
S:MMT0	2:1	98.64	347	7.1	99
	5:1	121.2	338	9.1	119
S:MMT1	2:1	105.5	299	17.8	143
	5:1	134.4	323	14.3	132
S:MMT2	2:1	97.1	330	8.6	102
	5:1	135.2	338	11.7	132
S:MMT3	2:1	111.8	318	12.3	114
	5:1	136.6	336	11.5	135

**Table 5:** The influence of the type and content of the biofiller used on the minimum M_min_ and the maximum M_max_ torque and its increase ΔM during vulcanization; standard deviations M_min_ ± 0.1 dNm, M_max_ ± 0.3 dNm, ΔM ± 0.3 dNm.

Sample Name	Weight Ratio of Straw to Montmorillonite	Filler Content [phr]	M_min_ [dNm]	M_max_ [dNm]	ΔM [dNm]
NR (Ref)		0	0.89	5.57	4.68
NR_S:MMT0	2:1	10	0.60	5.62	5.02
		20	0.82	6.41	5.59
		30	1.00	6.98	5.98
	5:1	10	0.48	6.03	5.55
		20	0.45	6.31	5.86
		30	0.72	7.6	6.88
NR_S:MMT1	2:1	10	1.02	8.97	7.95
		20	1.21	10.38	9.17
		30	1.26	11.27	10.01
	5:1	10	1.07	9.3	8.23
		20	1.14	10.9	9.76
		30	1.13	12.02	10.89
NR_S:MMT2	2:1	10	0.93	7.91	6.98
		20	0.91	10.41	9.5
		30	1.72	12.39	10.67
	5:1	10	0.88	7.75	6.87
		20	1.14	9.45	8.31
		30	1.17	10.54	9.37
NR_S:MMT3	2:1	10	1.13	8.11	6.98
		20	1.38	10.44	9.06
		30	1.81	11.6	9.79
	5:1	10	1.02	7.88	6.86
		20	1.31	9.22	7.91
		30	1.47	10.59	9.12

**Table 6:** Mechanical properties of vulcanizates under static tensile conditions; standard deviations: SE_100, 200, 300_ ± 0.2 MPa, E_b_ ± 15%.

Sample Name	Filler Content [phr]	Weight Ratio of Straw to Montmorillonite	SE_100_ [Mpa]	SE_200_ [Mpa]	SE_300_ [Mpa]	E_b_ [%]
NR (Ref)	0	-	0.75	1.12	1.53	692
NR_S:MMT0	10	2:1	0.82	1.27	1.75	700
	20		1.07	1.67	2.25	748
	30		1.27	1.92	2.51	724
	10	5:1	0.96	1.51	2.04	726
	20		1.07	1.74	2.34	718
	30		1.46	2.28	3.06	661
NR_S:MMT1	10	2:1	1.54	2.41	3.43	493
	20		1.91	2.86	4.14	497
	30		2.47	3.48	4.95	492
	10	5:1	1.58	2.44	3.44	714
	20		1.48	2.29	3.20	677
	30		2.45	3.39	4.68	545
NR_S:MMT2	10	2:1	1.22	1.97	2.75	650
	20		1.83	2.71	3.62	637
	30		2.64	3.73	5.48	511
	10	5:1	1.31	2.00	2.71	634
	20		1.80	2.59	3.41	615
	30		2.24	3.18	4.27	594
NR_S:MMT3	10	2:1	1.25	2.05	2.93	636
	20		1.72	2.70	3.82	584
	30		2.08	3.31	4.83	546
	10	5:1	1.27	2.00	2.74	627
	20		1.91	2.84	3.88	667
	30		2.52	3.50	4.84	617

## Data Availability

Data sharing not applicable.
